# Integrated ultrasonographic approach to evaluate fluid responsiveness in critically ill patients

**DOI:** 10.1038/s41598-023-36077-5

**Published:** 2023-06-06

**Authors:** Francesca Innocenti, Caterina Savinelli, Alessandro Coppa, Irene Tassinari, Riccardo Pini

**Affiliations:** High-Dependency Unit, Department of Clinical and Experimental Medicine, Azienda Ospedaliero-Universitaria Careggi, Lg. Brambilla 3, 50134 Florence, Italy

**Keywords:** Diseases, Medical research

## Abstract

In patients with acute circulatory failure, we tested the feasibility of the evaluation of the fluid-responsiveness (FR) by a combined approach with echocardiography and lung ultrasound. We enrolled 113 consecutive patients admitted to the Emergency Department High-Dependency Unit of Careggi University-Hospital from January 2015 to June 2020. We assessed: (1) inferior vena cava collapsibility index (IVCCI); (2) the variation of aortic flow (VTIAo) during the passive leg raising test (PLR); (3) the presence of interstitial syndrome by lung ultrasound. FR was defined as an increase in the VTIAo > 10% during PLR or IVCCI ≥ 40%. FR patients were treated with fluid and those non-FR with diuretics or vasopressors. The therapeutic strategy was reassessed after 12 h. The goal was to maintain the initial strategy. Among 56 FR patients, at lung ultrasound, 15 patients showed basal interstitial syndrome and 4 all-lung involvement. One fluid bolus was given to 51 patients. Among 57 non-FR patients, 26 patients showed interstitial syndrome at lung ultrasound (basal fields in 14, all lungs in 12). We administered diuretics to 21 patients and vasopressors to 4 subjects. We had to change the initial treatment plan in 9% non-FR patients and in 12% FR patients (p = NS). In the first 12 h after the evaluation, non-FR patients received significantly less fluids compared to those FR (1119 ± 410 vs 2010 ± 1254 ml, p < 0.001). The evaluation of the FR based on echocardiography and lung ultrasound was associated with the reduction in fluid administration for non-FR patients compared with those FR.

## Introduction

Optimal fluid management is one of the cornerstones of hemodynamic management in shock^1^. The basic physiological target of the administration of fluids is to improve tissue perfusion. In the earliest phase, especially during sepsis, a strong vasodilation leading to a low mean arterial pressure and microcirculatory impairment occurs, eventually associated with high or low cardiac output^2^. In this phase, the administration of fluids will significantly increase cardiac output in almost all cases, with a possible positive prognostic effect^3,4^. In the following phases, a condition of fluid responsiveness (FR) persists in less than half of the patients^1,5^. Therefore, a careful assessment of the potential benefits of fluids administration is highly advised^6^.

Current guidelines for the management of critical patients^1,7^ recommend the application of dynamic tests to evaluate fluid responsiveness, instead of static indices previously indicated, but the current practice and the modalities of evaluation of FR in critically ill patients are highly variable^8^. Moreover, the presence of fluid responsiveness should be balanced with the presence of fluid tolerance, that is the ability of the vasculature and organs to adsorb fluids.

Inferior vena cava collapsibility index (IVCCI) is one of the most popular method to evaluate FR, but its diagnostic accuracy has been questioned^9^. In non-ventilated patients, passive leg raising (PLR) associated with a non-invasive evaluation of cardiac output or aortic flow variation is actually one of the preferred options^10^. Lung ultrasound (LU) represents a feasible bedside tool to appreciate early signs of fluids overload in the lungs^11^, which represent one of the most important sites for the accumulation of extravascular fluid with consequent respiratory deterioration.

In patients with acute circulatory failure, we aimed to test the feasibility of the evaluation of the FR by a combined approach with echocardiography and lung ultrasound and the ability of this approach to improve the management of fluids administration.

## Materials and methods

### Study design and setting

The study protocol was approved by the “Toscana-Area Vasta-Centro” inter-institutional ethic committee (registration number CEAV 2018-484) and was conducted in accordance with the Helsinki Declaration of 1964 (revised 2008). All patients gave informed consent to enter the study.

In this interventional prospective study, we enrolled consecutive patients with acute circulatory failure (ACF) admitted to the Emergency Department High-Dependency Unit of Careggi University-Hospital in Florence from January 2015 to June 2020. All the patients had completed their initial resuscitation.

The ED-HDU (High-Dependency Unit) is a clinical setting where critical patients are managed, with availability of advanced monitoring, non-invasive ventilation and possibility to administer vasoactive drugs, managed by Emergency Physicians; all patients are admitted from the Emergency Department (ED), according to bed availability. Within 48 h from ED admission, the ED-HDU physicians must decide the optimal patients’ disposition, choosing between the ordinary ward, the intensive care unit or another HDU. Because our ED-HDU do not have invasive mechanical ventilators, patients already intubated in the Emergency Room or with a high probability of intubation in the first 24 h are directly admitted into the Intensive Care Unit^12^.

### Study population

We included patients who, after the completion of the early resuscitation, presented at least one of the following signs: (1) Systolic blood pressure < 90 mmHg or the need to use vasopressors, (2) Urine output < 0.5 ml/kg/h, (3) Persistent tachycardia (heart rate > 100 b/min in the absence of hyperthyroidism or fever, persisting more than 30 min) and (4) Mottled skin, as previously described by several authors^13–15^. Exclusion criteria were as follows: subjects aged < 18 years, pregnancy, cardiogenic or hemorrhagic shock, chronic kidney disease requiring dialysis, aortic valve disease (stenosis or regurgitation at least moderate), inadequate acoustic window.

For each patient, basic demographic data and clinical parameters were collected from medical records using a standardized collection template. Laboratory results were collected the day of the test and the day after.

### Study protocol

For each patient, we initially performed an echocardiographic examination, with a standardized protocol based on the recommendations of the American and European Societies of Echocardiography^16,17^. The exam included an assessment of dimensions and systolic function of the left (LV) and right ventricle (RV) and a comprehensive valvular assessment.

The inferior vena cava (IVC) was visualized from the subcostal window and the IVC collapsibility index (IVCCI) was calculated as [(Dmax − Dmin)/Dmax] × 100. An IVCCI ≥ 40% was the threshold to identify fluid-responder patients^18,19^.

For the passive leg raising, the patients were positioned for 10 min in a semirecumbent position (45°) and the echocardiographic assessment was performed. Then, using an automatic bed elevation technique, the patient’s trunk was lowered from the semirecumbent to the supine position, while the lower limbs were raised to a 45° angle and maintained in this position for 2 min. Patients were adequately sedated during the maneuver and vasoactive drugs, sedatives, and non-invasive ventilation parameters remained constant during the whole procedure^20^.

Using the apical five-chamber view, at baseline and during the leg raising, the velocity time integral of aortic blood flow (VTIAo) was computed from the area under the envelope of the pulsed-wave Doppler signal, obtained at the level of the aortic annulus. The VTIAo value was averaged over three consecutive measurements in patients with sinus rhythm and over five in those with atrial fibrillation. Patients were considered fluid-responders if the VTIAo increased ≥ 10% during the test. Patients were managed based on the results of PLR; if this test was not feasible, the therapeutic plan was decided based on IVCCI. In the event of persistent hemodynamic instability after the first fluid bolus, a reassessment was performed in order to evaluate the indication to administer further fluid boluses.

The lungs were examined by using longitudinal and oblique scans on anterolateral and posterior thoracic areas. Anterolateral examination was performed with the patient in the semirecumbent position; whenever possible, dorsal areas were scanned in the sitting position or by turning the patient in the lateral decubitus on both sides in case of forced supine position^21^.

B-lines were defined as hyperechoic, vertical artifacts arising from the pleural line, reaching the bottom of the screen, and possibly fading or obliterating A-lines. Interstitial syndrome (IS) was defined as signs in both right and left lungs involving 2 or more positive regions. The presence of an interstitial syndrome involving both basal and apical fields was considered a contraindication to the administration of fluids^11^. The results of the test were immediately available for treating physicians.

**Figure 1:**
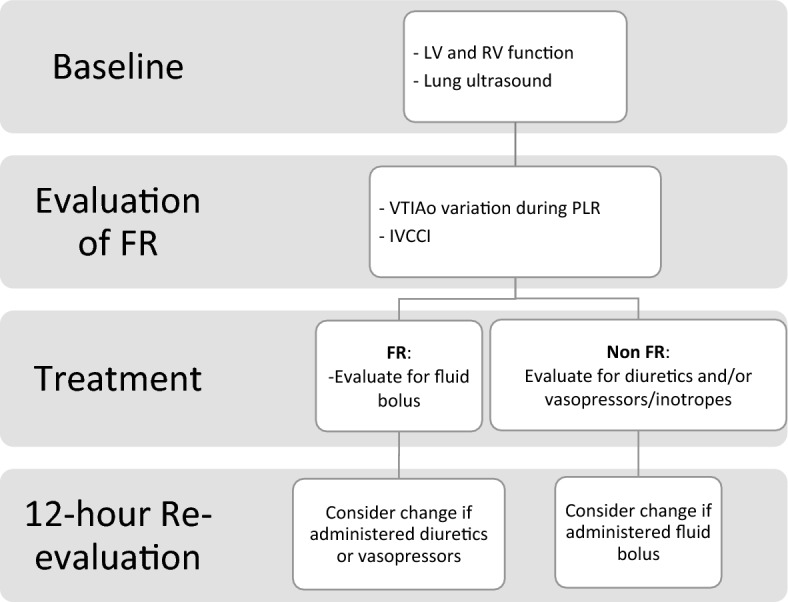
Study protocol.

The baseline assessment and management of FR and non-FR patients is depicted in Fig. 1. In FR patients, we considered the administration of a fluid bolus, which consisted of 500 ml of crystalloids (Ringer lactates). In non-FR patients, we considered the administration of diuretics or vasopressors The presence of FR was not considered a compulsory indication to administer a fluid bolus^20^ nor the absence of FR determined the administration of diuretics. The treatment based on the index evaluation was administered within the first hour and thereafter patients were managed according to their clinical condition. The goal was the maintenance of the therapeutic strategy planned after the initial evaluation over the following 12 h. We considered a failure of the initial evaluation the adoption, in the following 12 h, of one of the treatments not included in the arm initially chosen, namely the administration of diuretics in FR patients or the administration of fluids in non-FR patients.

### Statistical analysis

Statistical analyses were conducted using IBM SPSS software package (version 27). The calculation of the sample size was initially based on previous papers, which compared the diagnostic accuracy of PLR combined with echocardiography to evaluate FR. Given a sensitivity and specificity of the test around 90%^10^, confirmed in a preliminary analysis of the first 40 patients of this study group, the requested sample size was 12 patients. In the present study, we did not compare the test with a gold standard, but we assessed the correctness of our results with the following clinical management. As we did not find a study with a similar design for reference, we decided to include around one hundred patients.

Continuous variables were reported as mean ± standard deviation, and comparison between two groups was performed with the Student t-test for unpaired data. Categorical data were presented as absolute numbers and percentages and analyzed using contingency tables. Comparisons between two repeated measures was performed by the t-test for repeated measures.

The sensitivity, specificity, positive and negative predictive value (PPV and NPV) and accuracy of the tests were calculated according to standard formulas; comparison of these parameters between groups was made with Fisher exact test. A two-tails p value < 0.05 was considered significant.

## Results

Among 131 patients screened during the study period, 18 presented an exclusion criterion (12 with baseline inadequate acoustic window, 3 patients in dialysis and 3 with aortic valve disease). Therefore, the study population included 113 patients. Patients were included based on the following criteria: (1) 56 patients showed MAP < 65 mmHg, associated in 23 with tachycardia, in 11 with reduced urinary output and in 8 with both; (2) 45 patients showed reduced urinary output, in 14 associated with tachycardia; (3) 12 patients showed tachycardia, in the absence of other causes like fever or hyperthyroidism. At the first assessment, we performed the PLR in 93 patients and in 95 we evaluated IVCCI; 22 patients underwent a second evaluation by the PLR and 47 by IVCCI.

In Table 1, we reported the clinical characteristics of the study population, based on the status of fluid responsiveness. Anamnestic variables, as well as vital signs, were similar regardless of the presence of FR, except for a higher prevalence of CAD among non-FR patients, compared to those FR. At baseline, a similar proportion of FR and non-FR patients received vasoactive medications or non-invasive ventilation. The amount of the initial fluid bolus was similar in FR and non-FR patients, but the latter received a lower dose of maintenance infusion than the FR patients. LV dimensions and systolic function were similar regardless of the state of fluid responsiveness, while TAPSE was significantly lower in non-FR than FR patients (Table 2).

**Table 1:** Clinical and echocardiographic characteristics in the whole study population and in fluid-responder and non-fluid-responder patients.

	All (n = 113)	FR patients (n = 56)	Non-FR patients (n = 57)	p
Age (years)	74 ± 14	73 ± 14	75 ± 14	NS
Male gender (%)	64 (57%)	34 (61%)	30 (53%)	NS
Previous medical conditions				
Hypertension (%)	63 (56%)	31 (55%)	32 (56%)	NS
Diabetes (%)	27 (24%)	17 (30%)	10 (18%)	NS
COPD (%)	26 (23%)	11 (20%)	15 (26%)	NS
CKD (%)	20 (18%)	7 (13%)	13 (23%)	NS
CAD (%)	26 (23%)	7 (13%)	19 (33%)	0.006
Hepatic failure (%)	9 (8%)	4 (7%)	5 (9%)	NS
Neoplasia (%)	35 (32%)	17 (30%)	18 (32%)	NS
Vital signs				
RR (a/min)	23 ± 6	23 ± 6	23 ± 7	NS
HR (b/min)	95 ± 19	97 ± 21	94 ± 18	NS
SAP (mmHg)	112 ± 25	112 ± 26	112 ± 24	NS
MAP (mmHg)	76 ± 16	76 ± 17	75 ± 15	NS
GCS	14.5 ± 1.3	14.6 ± 1.0	14.5 ± 1.5	NS
Admission diagnosis				
Sepsis	87 (77%)	34 (61%)	53 (93%)	NS
COPD exacerbation	7 (6%)	6 (11%)	1 (2%)	NS
SOFA score	6 ± 3	6 ± 3	7 ± 4	NS
Lactate levels (mEq/L)	2.1 ± 2.4	2.1 ± 2.2	2.2 ± 2.5	NS
Noradrenaline infusion (%)	40 (35%)	19 (34%)	21 (37%)	NS
Non-invasive ventilation (%)	28 (25%)	15 (54%)	13 (56%)	NS
Pre-evaluation				
Fluid bolus (ml)	1101 ± 1096	1062 ± 1110	1140 ± 1090	NS
Fluids infusion (ml(h)	96 ± 42	110 ± 46	83 ± 33	< 0.001
Post-evaluation				
Total fluids	1561 ± 1028	2010 ± 1254	1119 ± 410	< 0.001

COPD: chronic obstructive pulmonary disease; CKD: chronic kidney disease; CAD: coronary artery disease; HR: heart rate; RR: respiratory rate; SAP: systolic arterial pressure; MAP: mean arterial pressure; GCS: Glasgow Come Scale; SOFA: sequential organ failure assessment.

**Table 2:** Echocardiographic parameters in the whole study population and in fluid-responder and non-fluid-responder patients.

	All (n = 113)	FR patients (n = 56)	Non-FR patients (n = 57)	p
LVDV (ml)	74 ± 31	77 ± 30	71 ± 32	NS
EF (%)	52 ± 13	54 ± 15	50 ± 12	NS
RV basal diameter (mm)	3.5 ± 0.9	3.3 ± 0.8	3.6 ± 0.9	NS
RV long. diameter (mm)	7.6 ± 1.1	7.5 ± 1.2	7.6 ± 1.1	NS
TAPSE (mm)	17 ± 25	19 ± 5	16 ± 5	0.023
LV syst. dysfunction (%)	40 (35%)	16 (29%)	24 (42%)	NS
RV syst. dysfunction (%)	38 (34%)	10 (18%)	28 (49%)	0.002

LVDV: left ventricular diastolic volume; RV: right ventricle; TAPSE: tricuspid annular plane systolic excursion.

**Figure 2:**
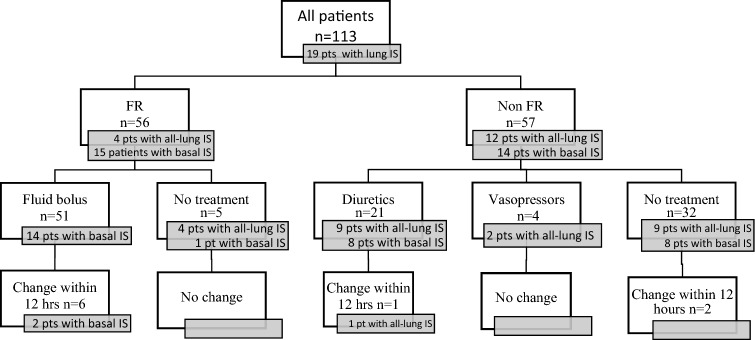
Management of FR and non-FR patients based on the integrated ultrasonographic evaluation. The treatment indicated in the third line have been added after the test.

In Fig. 2, we depicted the assessment and the treatment for FR and non-FR patients. Considering FR patients, in 51 the evaluation by the VTIAo variation during PLR was feasible and the treatment planning was based on it. In the remaining 5, the therapeutic decisions were based on IVCCI, which was the only available evaluation. Despite the condition of fluid responsiveness, 19 FR patients (34%) showed interstitial syndrome at lung ultrasound, which involved only basal fields in 15 and all lungs in 4. One fluid bolus was given to 51 patients, including 14 with basal interstitial syndrome and in 2 of them we needed to administer diuretics in following hours for worsening respiratory function; a second fluid bolus was given to 7 patients. In 5 patients, all with basal or all-lung IS, we did not administer a fluid bolus but we avoided diuretics and we could maintain the therapeutic strategy in the following hours. A change in the treatment strategy was necessary in 6 out of 51 patients (12%).

Among 57 non-FR patients, management was based on VTI variation during PLR in 42 patients and on IVCCI in 15. Twenty-six (46%) patients showed interstitial syndrome at lung ultrasound (p = NS vs FR patients), which involved only basal fields in 14 and all lungs in 12. Diuretics were administered to 21 patients and vasopressors were added to treatment in 4 subjects. A change in the treatment plan was made in 5 patients (9%, p = NS compared to FR patients). Therefore, considering both FR and non-FR patients, this approach allowed to establish a correct management of fluid administration in 102 out of 113 patients (90%). In the first 12 h after the evaluation, non-FR patients received significantly less fluids compared to those FR (Table 1).

**Figure 3:**
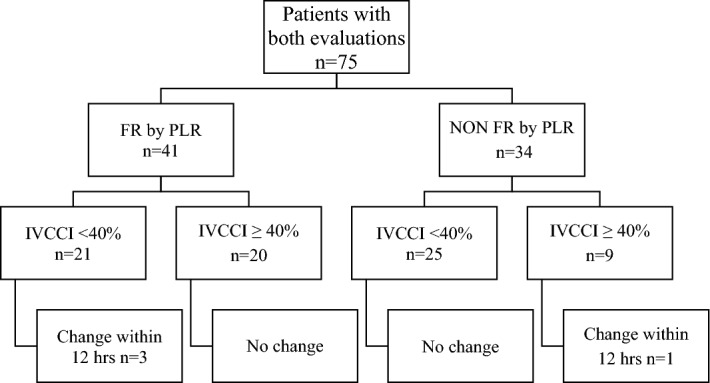
Comparison between VTIAo during PLR and IVCCI results.

**Figure 4:**
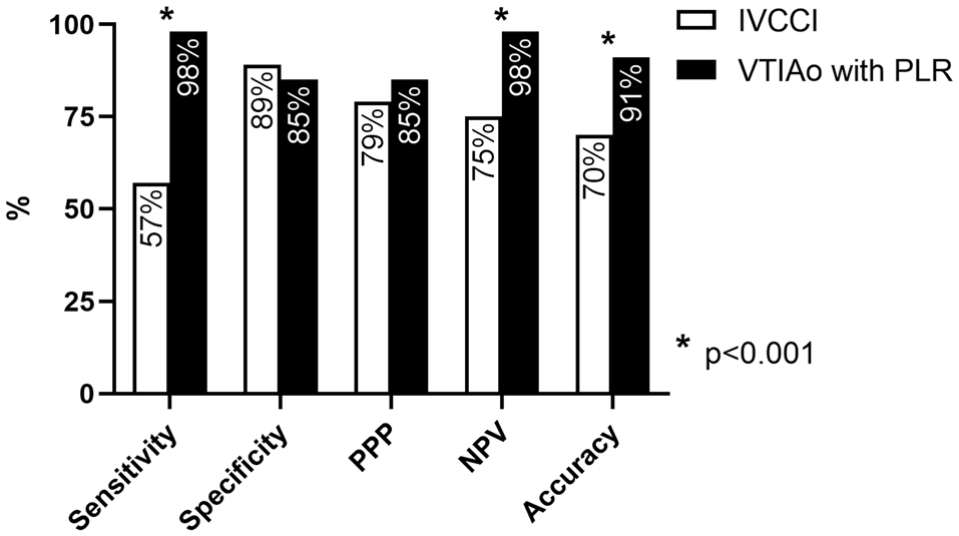
Diagnostic performance of VTIAo during PLR and IVCCI results.

Creatinine values demonstrated a significant reduction in the twelve hours following the FR assessment (1.8 ± 1.4 mg/dl vs 2. 0 ± 1.6 mg/dl, p = 0.031), while total bilirubin (1.22 ± 1.62 mg/dl vs 1.28 ± 1.93 mg/dl), alanine aminotransferase (102 ± 285 UI/L vs 76 ± 132 UI/L) and N-terminal brain natriuretic peptide (18,794 ± 32,645 ng/L vs 17,143 ± 32,531 ng/L, all p = NS) did not change. In Fig. 3, we compared the results of VTIAo and IVCCI in patients who performed both tests. We pooled the results of the first and the second evaluation to calculate the diagnostic performance of the two tests to evaluate FR and we reported them in Fig. 4. The diagnostic performance of the tests was evaluated based on their ability to identify the correct therapeutic strategy for the following 12 h. The evaluation of VTIAo during PLR showed a significantly better diagnostic accuracy than IVCCI. Both tests correctly identified a higher proportion of patients among non-FR (98% for VTIAo variation during PLR and 82% for IVCCI) than among FR patients (85% and 56%, respectively p = 0.019 and p = 0.002).

## Discussion

In a population of non invasive-ventilated patients with acute circulatory failure, vital signs did not discriminate between FR and non-FR patients. The combined approach involving echocardiography and lung ultrasound allowed a feasible and correct evaluation of the FR and the therapeutic plan based on this evaluation was maintained in most patients in the following 12 h. In patients evaluated by the variation of aortic flow during PLR, who accounted for the majority, the initial strategy was maintained more frequently than in those evaluated by the IVCCI. Anyway, as we decided to evaluate a protocol tailored for an HDU, where an invasive monitoring of cardiac output is not feasible, IVCCI represented a feasible alternative in patients who were not candidate to PLR.

Fluids are one of the most administered treatments in critically ill patients, but the awareness that they represent a real drug is not so widespread. In fact, both hypovolemia and hypervolemia are harmful states, with heavy consequence on the outcome of the patients, regardless of the cause of the shock state^6^. Both inadequate fluid replacement and fluid overload determine tissue hypoperfusion, the first for persistent low cardiac output and the latter for the presence of increased extravascular water, end-organ edema, with final organ dysfunction^2^. Fluid overload has been shown to be an independent predictor of mortality in the critically ills, including patients with septic shock, acute respiratory distress syndrome, and those undergoing major surgery^22–24^. To assess fluid responsiveness, dynamic tests are recommended over vital signs or static variables^25^. For non-ventilated patients, fluid challenge or PLR represents possible options as dynamic tests. PLR has the advantage of being completely reversible, as no fluids are administered; its drawbacks are represented by the limited accuracy in patients with high intra-abdominal pressure and the long appraisal curve in the event of using echocardiographic to monitor cardiac output^10^.

Several authors have demonstrated that a liberal approach to fluid therapy with positive fluid balance is associated with increased mortality rate^26,27^ and restrictive protocols have been proposed^28,29^. Douglas et coll.^30^ recently published the results of the FRESH protocol, which aimed to evaluate whether the resuscitation guided by the assessments of fluid responsiveness based on a dynamic test could improve patients’ outcome. The study was randomized and multicenter, but the study population was finally limited. In patients with septic shock, they demonstrated that fluid and vasopressor resuscitation guided by the use of dynamic tests was safe and determined a reduction in the risk of renal and respiratory failure requiring replacement treatments. These results were reached despite a modest difference in the total amount of fluids administered respectively in the intervention and the usual care arms, without any significant increase in creatinine values in the 72-h study period. Therefore, even a small excess of administered fluids seems to have relevant prognostic consequences.

Our study population included a mixed population of critically ill patients. We showed that a combined approach with the dynamic evaluation of FR associated with the assessment of extravascular lung water allowed us to correctly identify patients, who could benefit from volume replacement or removal. As recommended by the existing literature^20,31^, the presence or absence of FR was not considered an absolute indication to a fluid bolus or the administration of diuretics, but a guide to decide how to manage fluid administration and avoid the therapeutic measures indicated for the opposite condition. To the best of our knowledge, this is the first study, which assessed the FR with a combined evaluation of a dynamic index and LU. A condition of FR does not imply that the administration of a fluid bolus will improve the clinical status. Even in the presence of a cardiac output increase > 10%, it cannot be taken for granted that the oxygen delivery to peripheral tissues will increase^32^. Moreover, it has been demonstrated that the condition of FR markedly varies over time, even within few hours, and a careful monitoring is required to avoid fluids overload^33^. Another important element of novelty is the evaluation of the appropriateness of the therapy after 12 h from the initial evaluation. The treatment plan based on this evaluation was maintained in most patients and we did not observe negative effects on parameters of renal function, as a possible effect of an inadequate administration of fluids.

Among FR patients, a relevant proportion presented interstitial lung syndrome and the contemporary evaluation of both aspects prevented us to administer fluids in those with an extensive lung involvement. In critically ill patients, the presence of the pulmonary interstitial syndrome can be the sign of fluids overload, but can also represent an early indication of altered lung permeability in the context of acute lung injury^34,35^. This is especially true among septic patients, who represent a relevant proportion of this study population. In fact, despite the presence of FR, these patients were not fluid-tolerant and the risk of increasing extravascular lung water was high. The approach proposed in this study represents an option for serial evaluations at the bedside, as LU can give useful information on fluid tolerance, besides those on FR obtained by VTIAo variation during PLR.

The study has several limitations, first the single center design. We employed echocardiography and the determination of VTIAo variation, to evaluate the response to PLR. This technique requires an adequate training of the operators, thus limiting its applicability. The possibility to use different monitoring methods, like the bioreactance employed in the FRESH study, could improve the diffusion and the feasibility of the dynamic evaluation of FR. Secondly, we did not compare the results obtained with the echocardiographic evaluation of the FR with a reference method. The only possibility was an invasive method to measure the variation of the cardiac output during the maneuver, but this tool is not available in our clinical setting. Therefore, we decided to rely on the coherence of the clinical management with the results of the text in the 12 h following the text itself. In this way we were not able to evaluate the accuracy of the method, but anyway we tested the correctness of our evaluation. We are also aware that the FR status of critical patients may vary, but we considered 12 h an adequate time interval to maintain the initial management plan. On the other side, only a randomized blinded study could allow us to evaluate the impact of this strategy on strong outcomes, like the reduction of in-hospital mortality as well as hospital length of stay. Finally, we are aware that treating physicians were not blinded to the results of the FR evaluation and could have been influenced in their clinical choices by those results. However, the staff was clearly informed about the study design and the treatment strategy was decided on a clinical basis.

## Conclusions

In a group of non-ventilated patients, who had already undergone the initial resuscitation, we demonstrated that the evaluation of the FR based on echocardiography and lung ultrasound increased the physician's confidence in reducing the amount of fluids in the patient judged as non-responders, without negative effects on renal function parameters.

## Data Availability

The datasets used and analyzed during the current study is available from the corresponding author (F. Innocenti, innocenti.fra66@gmail.com) on reasonable request.
